# Construction of China national newborn growth standards based on a large low-risk sample

**DOI:** 10.1038/s41598-021-94606-6

**Published:** 2021-08-09

**Authors:** Xin-Nan Zong, Hui Li, Ya-Qin Zhang, Hua-Hong Wu, Geng-Li Zhao, The Working Committee of the China National Newborn Growth Standards Project, Hui Li, Ya-Qin Zhang, Xin-Nan Zong, Hua-Hong Wu, Geng-Li Zhao, Qi Feng, Dan-Hua Wang, Ying Pan, Hui-Juan Yang, Bo-Zhi Lu, Ya-Jing Guo, Xiao-Mei Xiang, Min Dong, Jing Zhang, Mei Wei, Zhang-Bin Yu, Shu-Ping Han, Ai-Fen Zhou, Ya-Qi Zhang, Yong Guo, Xian Liu, Pin Ge, Fang Guo, Jun Zheng, Xiu-Ying Tian, Bei Lin, Xiao-Mei Qiu, Shao-Jie Yue

**Affiliations:** 1 Department of Growth and Development, Capital Institute of Pediatrics, No.2 Yabao Road, Chaoyang District, Beijing, 100020 China; 2 Department of Gynecology and Obstetrics, Peking University First Hospital, No.8 Xishiku Street, Xicheng District, Beijing, 100034 China; 3 Capital Institute of Pediatrics, Beijing, China; 4 Peking University First Hospital, Beijing, China; 5 Peking Union Medical College Hospital, Beijing, China; 6 Beijing Obstetrics and Gynecology Hospital, Capital Medical University, Beijing, China; 7 Harbin Maternal and Child Health and Family Planning Service Center, Harbin, China; 8 Xi’an Maternal and Child Health Hospital, Xi’an, China; 9 Shanghai Center for Women and Children’s Health, Shanghai, China; 10 Women’s Hospital of Nanjing Medical University, Nanjing, China; 11 Wuhan Maternal and Child Health Care Hospital, Wuhan, China; 12 Guangzhou Women and Children’s Medical Center, Guangzhou, China; 13 Fujian Maternal and Child Health Care Hospital, Fujian, China; 14 Kunming City Maternal and Child Health Hospital, Kunming, China; 15 Tianjin Central Hospital of Gynecology Obstetrics, Tianjin, China; 16 Shengjing Hospital of China Medical University, Shengjing, China; 17 Shenzhen Maternal & Child Healthcare Hospital, Shenzhen, China; 18 Xiangya Hospital Central South University, Xiangya, China

**Keywords:** Paediatric research, Neonatology, Preterm birth

## Abstract

Most published newborn growth references are based on conventional monitoring data that usually included both low- and high-risk pregnancies. We sought to develop a set of neonatal growth standards constructed from only a large sample of low-risk pregnancies. A total of 24,375 naturally conceived singleton live births with gestational ages of 24–42 weeks were collected in 69 hospitals in thirteen Chinese cities between 2015 and 2018. Unhealthy infants or those with high-risk mother were excluded. Smoothed percentile curves of six anthropometric indicators were established using the Generalized Additive Model for Location, Scale and Shape. The 3rd, 10th, 25th, 50th, 75th, 90th, and 97th percentile references for birth weight, length, head circumference, weight/length, body mass index, and ponderal index were calculated for neonates with gestational ages of 24–42 weeks. This set of neonatal growth standards with six anthropometric indicators can provide more tools for growth and nutrition assessment and body proportionality in neonatal clinical practice. These standards might also help to show the differences between growth curves based on low-risk and mixed low- and high-risk pregnancies.

## Introduction

Intrauterine growth is associated with infant survival, future growth and development, and health conditions. An appropriate growth standard or reference is an essential tool for neonatal growth assessment at birth. Therefore, some countries have established newborn growth standards based on low-risk pregnancies with a normal outcome^1,2^ or newborn growth references based on mixed low- and high-risk pregnancies^3–6^. Whether sample population are drawn from low-risk pregnancies or mixed low- and high-risk pregnancies is the key to distinguish growth standards and growth references^7,8^. The sample populations for growth standards are selected based on relatively healthy and adequately nourished pregnant women and represent relative healthy patterns of growth of neonates that can answer how infants ought to grow rather than how they do grow^9^. Considering the difference of the reference samples, a standard may have more clinical utility than a population reference^8^.

The growth reference for Chinese newborns constructed in 1988^10^ is ill-suited for assessing the growth and development of today’s Chinese newborns due to several limitations. Recent monitoring data has shown that the growth level of newborns is substantially higher compared to the 1988 reference^11–13^. Further, the 1988 reference only covers newborns with a gestational age (GA) of greater than 28 weeks, which does not meet the current need of assessing infants with a GA of less than 28 weeks as more extremely preterm babies are being born. To overcome these limitations, we aimed to develop a set of growth standards for neonates with GA of 24–42 weeks based on a large sample of low-risk pregnancies. Three commonly used indicators—birth weight, length, and head circumference—that allowed better definition of small for GA (SGA) and large for GA (LGA) were employed. In addition, three anthropometric ratios—weight/length, body mass index (BMI), and ponderal index (PI)—that allowed for better assessment of symmetric vs asymmetric abnormalities in growth were utilized. We also examined the differences among our standards, the INTERGROWTH-21st standards^1,14,15^ and the new US curves^4,16^.

## Materials and methods

### Subject

From June 2015 to November 2018, a cross-sectional survey of newborn babies with a GA of 24–42 weeks in 13 cities in China was carried out. Of these 13 cities, nine, including Beijing, Harbin, Xi’an, Shanghai, Nanjing, Wuhan, Guangzhou, Fuzhou, and Kunming, were selected to investigate newborn babies with GA of 24–42 weeks. These nine cities were also the sample cities on the National Survey on the Physical Growth and Development of Children in China, which was a representative national survey of the country^17^. Considering the low numbers of extremely and early preterm babies, four cities in the surrounding regions of the nine cities including Tianjin, Shenyang, Changsha, and Shenzhen were added to supplement the sample sizes of preterm babies with GA of 32 weeks and below.

Single naturally conceived live births with a GA of 24–42 weeks were included. Infants who were not healthy or whose mothers were at high health risk were excluded according to the following exclusion criteria: ① unclear GA; ② severe congenital malformation at birth or known chromosomal abnormality; ③ edema or hematoma during physical measurement; ④ parents of non-Chinese origin; ⑤ mothers were not permanent residents in surveyed cities and lived in surveyed cities for < 2 years; ⑥ maternal height < 145 cm; ⑦maternal age < 18 years or > 40 years; ⑧ mothers who were smoking, alcoholic or drug dependent over the three months before or during pregnancy; ⑨ mothers who had continuously taken adrenal cortex hormones or other immunosuppressive agents for > 1 month during pregnancy; ⑩ mothers of full-term babies with any of the following conditions during pregnancy: severe anemia (Hb ≦ 60 g/L), gestational diabetes, preeclampsia, eclampsia, hyperthyroidism or hypothyroidism, heart and kidneys dysfunction, chronic hypertension; ⑪ mothers of preterm babies with any of the following conditions during pregnancy: severe anemia (Hb ≦ 60 g/L), gestational diabetes that cannot be effectively controlled by diet and exercise intervention, severe preeclampsia, eclampsia, hyperthyroidism or hypothyroidism that cannot be effectively controlled by drug therapy, severe heart and kidneys dysfunction.

GA was jointly determined based on the mother’s last menstrual period (LMP) and the results of ultrasound examination in the first three months of pregnancy. GA based on LMP was used when the GA difference between the two methods was ≦ 1 week, and GA based on ultrasound examination was used when the GA difference was > 1 week. GA groups were divided by week, such as 24^+0^ to 24^+6^ weeks for the 24 week GA group.

Sample size was estimated according to both statistical accuracy requirements for establishing percentile curves and observed numbers of newborns at each GA. For full-term babies with GA of 37–41 weeks, the sample size for each GA group was about 100 per sex and city; for preterm babies with GA of 29–36 weeks, the sample size for each GA group was about 50. Full-term babies with GA of 37–41 weeks were sampled by cluster sampling. Samples were evenly distributed by season, and babies randomly selected from each season. All eligible full-term babies with GA ≧ 42 weeks or preterm babies with GA ≦ 28 weeks within selected hospitals were included in this study. The study was reviewed and approved by the Ethics Committees of the Capital Institute of Pediatrics (No.SHERLL-2015009). Written informed consent at the top of questionnaires was obtained from all respondents (i.e., parents of the newborns). All methods were performed in accordance with the relevant guidelines and regulations.

### Measurement

Birth weight was measured within 12 h of birth with an electronic scale (maximum range 20 kg, accurate to 10 g). Birth recumbent length was measured within 24 h with infantometer (maximum range 65 cm, accurate to 0.1 cm) for term babies and preterm babies of large GA and new patent infantometer (Patent No. zl201520996396.X) (maximum range 45 cm, accurate to 0.1 cm) for preterm babies of small GA. Birth head circumference was measured within 24 h with a flexible non-stretchable plastic tape (0.7 cm wide, maximum range 100 cm, accurate to 0.1 cm). Birth weight, length, and head circumference were measured twice and recorded twice in a standardized measurement procedure^18^. Each measurement was collected independently by two trained doctors or nurses. If the difference between the two measurements exceeded the maximum allowable difference (weight 10 g, length 0.5 cm, head circumference 0.5 cm), a third measurement was taken, and then those two measurements not exceeding the allowable difference were recorded.

### Quality control

Uniform measuring tools were equipped for all sites, including infantometer and new patent infantometer for length measurement, non-stretchable plastic tape for head circumference measurement, standard weights (accuracy 10 g, 50 g, 100 g, 500 g) for calibration of electronic scale, and standard steel tape (accuracy 1 mm) for calibration of infantometer and plastic tape. The electronic scales in each site were used for investigation after evaluation and calibration of standard weights with a maximum allowable difference of 10 g. Calibration was taken every week with maximum allowable differences of weight 10 g, length 0.5 cm, and head circumference 0.5 cm. Questionnaires were completed by pairs of trained doctors or nurses, with one recording the answers and the other reviewing. The completed questionnaires in each city were sent to the Beijing Steering Committee for final check and data entry. EpiData 3.0 software was used for double entry and logic check of the questionnaires.

### Statistical analysis

The mean of two measurements for birth weight, length and head circumference was used for data analysis and calculation of anthropometric ratio. Weight/length, BMI, and PI were calculated according to the following formula: [weight (kg)/length (m)], [weight (kg)/length (m)^2^], and [weight (kg)/length (m)^3^], respectively. During data cleaning, we excluded 2 missing weight values, 8 missing length values, and 16 missing head circumference values. Few measures not within ± 5 standard deviation (SD) of the mean of overall sex- and GA-specific values was also excluded (12 for weight, 17 for length, 10 for head circumference, 29 for weight/length, 49 for BMI, and 208 for PI). The final sample sizes contributing to the establishment of the growth curves for each indicator are listed below: weight (13,192 males and 11,169 females), length (13,183 and 11,167), head circumference (13,181 and 11,168), weight/length (13,176 and 11,159), BMI (13,162 and 11,153), and PI (13,075 and 11,081). During the establishment of the growth curves, normality test, and skewness and kurtosis analysis were assessed for each indicator. Data analyses used SAS v9.4 (SAS Institute Inc).

The Generalized Additive Model for Location, Scale and Shape (GAMLSS) which is a general framework for fitting regression models where the distribution of the response variable allows for highly skewed and kurtotic continuous distribution^19^, was employed to establish smoothed percentile growth curves of male and female newborn babies with GA of 24–42 weeks. Curve fitting was performed using the GAMLSS 4.3–1 library running under R 3.1.2. Goodness of fit of the GAMLSS models was assessed by the Schwarz Bayesian Information Criterion that is justified as a general criterion for model selection and by Q-Q plots that assesses the age-conditional normality of the transformed data^20,21^. After comparative testing of alternative methods (ie, distribution transformation and smoothing function) used to generate the growth curves, birth weight, weight/length, BMI, and PI percentile curves were established using the GAMLSS with Box-Cox *t* (BCT) distribution with cubic splines, and birth length and head circumference using the GAMLSS with Box-Cox power exponential (BCPE) distribution with cubic spline. All these GAMLSS models did not need to be weighted because the difference of each indicator between non-weighting and equal proportional weighting were negligibly small^16^. The differences between fitted percentiles and empirical values at each week were examined for all the six anthropometric indicators.

## Results

### Basic characteristics of the reference sample

A total of 24,375 singleton live births with GA of 24–42 weeks were collected in 69 hospitals in 13 Chinese cities, including 12,264 preterm babies (7,042 males and 5,222 females) and 12,111 full-term babies (6,155 males and 5,956 females). Table 1 presents sample sizes and means (SD) of birth weight, length, and head circumference by sex and GA. Table 2 presents sample sizes of the 13 cities by city and GA. 61.4% of newborn babies were delivered vaginally and 38.6% by cesarean section. The proportion for first births was 65.9% and for second and higher 34.1%. Mothers with high school and college degrees accounted for 84.8% of the sample population, and childbearing age was 31.5 (± 5.0) years. Average maternal height was 161.0 (± 5.0) cm, pre-pregnancy BMI was 21.0 (± 3.0) kg/m^2^, and weight gain during pregnancy was 13.8 (± 5.0) kg.

**Table 1:** Sample size, and mean (SD) of birth weight, length and head circumference, by sex and GA.

GA (weeks)	Male				Female			
	Sample size	Weight	Length	Head circumference	Sample size	Weight	Length	Head circumference
24	26	708.3 (161.7)	32.1 (2.9)	22.2 (2.6)	15	664.7 (187.1)	31.6 (2.6)	21.3 (2.1)
25	40	875.7 (134.3)	34.6 (3.0)	24.1 (2.1)	17	814.4 (205.0)	32.3 (3.1)	23.0 (2.7)
26	79	969.0 (132.0)	34.6 (3.1)	24.8 (1.8)	40	877.8 (112.2)	34.3 (3.0)	24.5 (1.9)
27	136	1107.6 (185.7)	36.6 (2.8)	25.6 (1.9)	106	987.0 (174.2)	35.0 (3.0)	25.2 (1.9)
28	305	1215.7 (210.4)	37.5 (2.9)	26.6 (1.8)	212	1161.1 (200.6)	37.2 (2.9)	26.6 (1.9)
29	353	1344.7 (234.4)	38.5 (2.8)	27.4 (1.8)	279	1260.4 (198.0)	38.3 (3.0)	26.7 (1.9)
30	497	1499.9 (257.7)	40.3 (2.7)	28.4 (1.8)	356	1430.6 (256.8)	39.6 (2.8)	27.9 (1.9)
31	631	1681.5 (260.3)	41.6 (2.8)	29.0 (1.7)	457	1568.3 (291.1)	40.9 (2.8)	28.6 (1.9)
32	774	1874.7 (313.8)	43.0 (2.7)	29.8 (1.8)	516	1773.7 (310.5)	42.2 (2.7)	29.4 (1.9)
33	714	2118.2 (324.0)	44.6 (2.5)	30.9 (1.8)	498	1965.6 (353.1)	43.5 (2.8)	30.2 (1.9)
34	948	2328.0 (353.9)	45.7 (2.4)	31.6 (1.6)	710	2216.0 (335.5)	44.9 (2.5)	31.1 (1.7)
35	1085	2569.3 (389.3)	47.0 (2.2)	32.2 (1.6)	910	2453.4 (384.8)	46.3 (2.2)	31.8 (1.7)
36	1454	2798.2 (390.0)	48.1 (2.1)	32.7 (1.5)	1106	2672.0 (390.5)	47.5 (2.2)	32.3 (1.6)
37	1020	3087.7 (345.3)	49.5 (1.5)	33.3 (1.3)	857	2956.3 (363.7)	48.9 (1.7)	32.9 (1.4)
38	1234	3284.0 (376.6)	50.0 (1.5)	33.8 (1.4)	1210	3156.8 (356.7)	49.4 (1.5)	33.4 (1.3)
39	1549	3389.7 (369.2)	50.5 (1.5)	34.0 (1.3)	1440	3271.5 (355.2)	49.9 (1.5)	33.7 (1.3)
40	1380	3499.5 (375.7)	50.9 (1.5)	34.2 (1.4)	1377	3377.6 (379.8)	50.4 (1.4)	33.8 (1.3)
41	926	3574.6 (378.6)	51.1 (1.5)	34.4 (1.4)	1006	3474.2 (360.6)	50.6 (1.4)	34.1 (1.4)
42	46	3570.8 (472.7)	51.2 (1.8)	34.4 (1.3)	66	3501.8 (352.6)	50.8 (1.6)	34.4 (1.6)

**Table 2:** Sample size contributing to this study, by city and GA.

Surveyed city	GA (weeks)				Total
	24–28	29–32	33–36	37–42	
Beijing	125	388	824	1113	2450
Harbin	50	215	758	1079	2102
Xi’an	94	416	905	1761	3176
Shanghai	55	178	545	971	1749
Nanjing	189	728	984	1565	3466
Wuhan	117	767	731	1084	2699
Guangzhou	94	351	1293	1955	3693
Fuzhou	56	252	735	995	2038
Kunming	82	347	649	1588	2666
Tianjin	47	75	NA	NA	122
Shenyang	11	55	NA	NA	66
Changsha	13	46	NA	NA	59
Shenzhen	43	46	NA	NA	89
Total	976	3864	7424	12,111	24,375

### Percentile reference values of six anthropometric indicators

Tables 3, 4, 5, 6, 7 and 8 present the 3rd, 10th, 25th, 50th, 75th, 90th, and 97th percentile reference values of weight for GA, length for GA, head circumference for GA, weight/length for GA, BMI for GA, and PI for GA for male and female newborns with GA of 24–42 weeks. All the six anthropometric indicators increased rapidly with GA, but growth velocity decreased slightly after 37 weeks.

**Table 3:** Percentile reference values of birth weight (in g) for Chinese newborn infants aged 24–42 weeks.

GA (weeks)	Male							Female						
	P_3_	P_10_	P_25_	P_50_	P_75_	P_90_	P_97_	P_3_	P_10_	P_25_	P_50_	P_75_	P_90_	P_97_
24	455	570	655	732	804	874	959	416	498	564	629	692	756	833
25	513	640	734	819	900	978	1072	479	572	648	722	796	869	958
26	580	719	823	918	1008	1096	1200	549	654	741	826	911	995	1096
27	657	809	924	1030	1130	1228	1343	626	745	843	941	1038	1135	1250
28	745	910	1036	1154	1267	1375	1503	711	844	955	1067	1178	1288	1418
29	845	1023	1162	1293	1418	1539	1680	804	951	1076	1203	1330	1455	1601
30	958	1150	1302	1446	1586	1720	1876	906	1068	1209	1352	1495	1636	1800
31	1087	1292	1457	1617	1771	1920	2091	1020	1198	1354	1515	1676	1835	2018
32	1233	1451	1630	1805	1976	2140	2328	1151	1344	1516	1694	1875	2051	2254
33	1400	1628	1820	2012	2199	2380	2585	1302	1509	1696	1892	2091	2285	2506
34	1586	1823	2027	2234	2438	2634	2856	1477	1695	1896	2108	2323	2534	2771
35	1791	2033	2247	2467	2686	2897	3133	1676	1902	2113	2338	2568	2791	3042
36	2015	2258	2477	2707	2937	3159	3406	1896	2125	2342	2575	2815	3047	3305
37	2247	2487	2708	2943	3181	3410	3664	2130	2357	2574	2810	3052	3287	3546
38	2468	2701	2921	3157	3399	3632	3889	2358	2579	2792	3026	3266	3498	3753
39	2649	2874	3091	3329	3573	3809	4068	2547	2762	2971	3202	3440	3670	3920
40	2783	3002	3216	3455	3702	3941	4203	2686	2896	3104	3336	3575	3806	4055
41	2886	3100	3314	3554	3806	4051	4319	2796	3005	3214	3448	3691	3925	4178
42	2977	3188	3402	3647	3907	4161	4438	2891	3101	3312	3551	3801	4042	4301

**Table 4:** Percentile reference values of birth length (in cm) for Chinese newborn infants aged 24–42 weeks.

GA (weeks)	Male							Female						
	P_3_	P_10_	P_25_	P_50_	P_75_	P_90_	P_97_	P_3_	P_10_	P_25_	P_50_	P_75_	P_90_	P_97_
24	26.9	28.3	29.7	31.2	32.6	33.8	35.0	26.9	28.2	29.4	30.6	31.8	32.8	33.7
25	28.1	29.6	31.0	32.5	34.0	35.3	36.5	28.0	29.4	30.6	32.0	33.2	34.2	35.2
26	29.2	30.8	32.3	33.9	35.4	36.7	38.0	29.1	30.6	31.9	33.3	34.7	35.8	36.8
27	30.5	32.1	33.7	35.3	36.9	38.3	39.6	30.2	31.8	33.2	34.7	36.2	37.4	38.5
28	31.7	33.4	35.1	36.8	38.4	39.8	41.2	31.4	33.0	34.6	36.2	37.7	39.0	40.2
29	33.0	34.8	36.5	38.2	39.9	41.3	42.7	32.5	34.3	35.9	37.6	39.2	40.5	41.8
30	34.3	36.2	37.9	39.7	41.4	42.8	44.2	33.8	35.6	37.3	39.0	40.7	42.1	43.4
31	35.7	37.7	39.4	41.2	42.8	44.3	45.6	35.1	36.9	38.6	40.4	42.1	43.5	44.9
32	37.2	39.1	40.9	42.6	44.3	45.6	47.0	36.4	38.3	40.0	41.8	43.5	44.9	46.3
33	38.7	40.7	42.4	44.1	45.6	46.9	48.3	37.8	39.7	41.4	43.2	44.9	46.3	47.6
34	40.2	42.2	43.8	45.4	46.8	48.2	49.5	39.3	41.2	42.9	44.6	46.2	47.5	48.7
35	41.8	43.6	45.2	46.6	48.0	49.2	50.7	40.8	42.7	44.3	45.9	47.4	48.6	50.0
36	43.2	45.0	46.4	47.7	49.0	50.4	51.8	42.4	44.1	45.7	47.1	48.5	49.6	50.9
37	44.4	46.2	47.5	48.7	49.8	51.2	52.9	43.7	45.3	46.9	48.2	49.4	50.4	51.9
38	45.6	47.3	48.5	49.5	50.6	52.1	53.7	44.8	46.4	47.9	49.1	50.1	51.1	52.6
39	46.5	48.2	49.3	50.3	51.2	52.6	54.4	45.8	47.3	48.7	49.9	50.7	51.7	53.2
40	47.3	48.9	49.8	50.8	51.7	53.1	54.9	46.5	48.1	49.4	50.4	51.3	52.3	53.7
41	47.9	49.4	50.2	51.2	52.1	53.5	55.3	47.1	48.7	49.8	50.9	51.7	52.6	54.2
42	48.3	49.7	50.5	51.4	52.4	53.8	55.6	47.6	49.2	50.1	51.2	52.0	53.0	54.5

**Table 5:** Percentile reference values of head circumference (in cm) for Chinese newborn infants aged 24–42 weeks.

GA (weeks)	Male							Female						
	P_3_	P_10_	P_25_	P_50_	P_75_	P_90_	P_97_	P_3_	P_10_	P_25_	P_50_	P_75_	P_90_	P_97_
24	19.4	20.3	21.2	22.0	22.8	23.5	24.0	19.3	20.0	20.7	21.6	22.3	22.8	23.2
25	20.3	21.3	22.2	23.1	23.9	24.6	25.2	20.1	20.9	21.7	22.6	23.3	23.9	24.4
26	21.2	22.2	23.2	24.1	25.0	25.7	26.4	20.9	21.8	22.6	23.6	24.4	25.0	25.6
27	22.1	23.2	24.1	25.1	26.0	26.8	27.5	21.7	22.7	23.6	24.5	25.4	26.1	26.7
28	23.0	24.1	25.1	26.1	27.0	27.8	28.6	22.6	23.5	24.5	25.5	26.5	27.2	27.9
29	23.9	25.0	26.0	27.0	28.0	28.9	29.7	23.4	24.4	25.4	26.5	27.5	28.3	29.0
30	24.7	25.8	26.9	28.0	29.0	29.9	30.7	24.2	25.2	26.3	27.4	28.5	29.3	30.1
31	25.6	26.7	27.7	28.8	29.9	30.8	31.7	25.0	26.1	27.2	28.3	29.4	30.3	31.1
32	26.4	27.5	28.6	29.7	30.7	31.7	32.6	25.9	27.0	28.1	29.2	30.3	31.2	32.1
33	27.3	28.4	29.4	30.5	31.5	32.5	33.4	26.8	27.9	28.9	30.1	31.1	32.1	33.0
34	28.1	29.2	30.2	31.3	32.3	33.2	34.2	27.7	28.7	29.7	30.8	31.9	32.8	33.7
35	28.9	30.0	30.9	31.9	32.9	33.9	34.8	28.5	29.5	30.5	31.5	32.6	33.5	34.4
36	29.7	30.6	31.6	32.5	33.5	34.4	35.3	29.3	30.2	31.2	32.2	33.1	34.0	34.9
37	30.3	31.2	32.1	33.1	34.0	34.9	35.8	30.0	30.9	31.8	32.7	33.6	34.5	35.3
38	30.9	31.8	32.6	33.5	34.4	35.3	36.1	30.5	31.4	32.3	33.1	34.0	34.8	35.7
39	31.3	32.2	33.0	33.9	34.7	35.6	36.5	31.0	31.9	32.7	33.5	34.3	35.2	36.0
40	31.6	32.5	33.3	34.1	35.0	35.8	36.7	31.4	32.2	33.0	33.8	34.6	35.4	36.3
41	31.9	32.8	33.6	34.4	35.2	36.0	36.9	31.7	32.5	33.3	34.1	34.9	35.7	36.6
42	32.2	33.0	33.8	34.6	35.4	36.2	37.1	31.9	32.8	33.6	34.3	35.2	36.0	36.9

**Table 6:** Percentile reference values of birth weight/length (in kg/m) for Chinese newborn infants aged 24–42 weeks.

GA (weeks)	Male							Female						
	P_3_	P_10_	P_25_	P_50_	P_75_	P_90_	P_97_	P_3_	P_10_	P_25_	P_50_	P_75_	P_90_	P_97_
24	1.5	1.8	2.1	2.3	2.5	2.7	2.9	1.6	1.8	2.0	2.2	2.4	2.6	2.8
25	1.7	2.0	2.2	2.5	2.7	2.9	3.1	1.7	1.9	2.1	2.4	2.6	2.8	3.0
26	1.8	2.2	2.4	2.7	2.9	3.1	3.3	1.8	2.1	2.3	2.5	2.8	3.0	3.2
27	2.0	2.4	2.6	2.9	3.1	3.4	3.6	2.0	2.2	2.5	2.7	3.0	3.2	3.5
28	2.2	2.6	2.8	3.1	3.4	3.6	3.9	2.1	2.4	2.7	2.9	3.2	3.5	3.7
29	2.4	2.8	3.1	3.4	3.6	3.9	4.2	2.3	2.6	2.9	3.2	3.5	3.7	4.0
30	2.7	3.0	3.3	3.6	3.9	4.2	4.5	2.5	2.8	3.1	3.4	3.8	4.0	4.4
31	2.9	3.3	3.6	3.9	4.2	4.5	4.9	2.8	3.1	3.4	3.7	4.1	4.4	4.7
32	3.2	3.6	3.9	4.2	4.6	4.9	5.2	3.0	3.4	3.7	4.0	4.4	4.7	5.1
33	3.5	3.9	4.2	4.6	4.9	5.2	5.6	3.3	3.7	4.0	4.4	4.7	5.1	5.5
34	3.8	4.2	4.6	4.9	5.3	5.6	6.0	3.6	4.0	4.4	4.7	5.1	5.5	5.9
35	4.1	4.5	4.9	5.3	5.7	6.0	6.4	3.9	4.3	4.7	5.1	5.5	5.9	6.3
36	4.5	4.9	5.3	5.7	6.1	6.4	6.8	4.3	4.7	5.1	5.5	5.9	6.3	6.7
37	4.8	5.2	5.6	6.0	6.4	6.8	7.2	4.7	5.1	5.4	5.8	6.2	6.6	7.1
38	5.2	5.6	5.9	6.4	6.8	7.2	7.6	5.0	5.4	5.8	6.2	6.6	7.0	7.4
39	5.5	5.8	6.2	6.6	7.0	7.4	7.9	5.3	5.7	6.0	6.4	6.9	7.2	7.7
40	5.7	6.0	6.4	6.8	7.2	7.6	8.0	5.5	5.9	6.2	6.6	7.1	7.4	7.9
41	5.8	6.2	6.6	7.0	7.4	7.8	8.2	5.7	6.1	6.4	6.8	7.2	7.6	8.0
42	6.0	6.4	6.7	7.1	7.5	7.9	8.3	5.9	6.2	6.6	7.0	7.4	7.8	8.2

**Table 7:** Percentile reference values of birth BMI (in kg/m^2^) for Chinese newborn infants aged 24–42 weeks.

GA (weeks)	Male							Female						
	P_3_	P_10_	P_25_	P_50_	P_75_	P_90_	P_97_	P_3_	P_10_	P_25_	P_50_	P_75_	P_90_	P_97_
24	5.1	5.8	6.5	7.1	7.8	8.5	9.4	4.9	5.5	6.1	6.8	7.5	8.2	9.1
25	5.4	6.1	6.7	7.4	8.1	8.8	9.7	5.2	5.8	6.4	7.1	7.8	8.5	9.4
26	5.6	6.4	7.0	7.7	8.4	9.1	10.0	5.5	6.1	6.7	7.4	8.1	8.9	9.8
27	5.9	6.7	7.3	8.0	8.7	9.5	10.4	5.8	6.4	7.0	7.7	8.5	9.2	10.1
28	6.3	7.0	7.6	8.3	9.1	9.8	10.7	6.1	6.7	7.4	8.1	8.8	9.6	10.5
29	6.6	7.3	8.0	8.7	9.4	10.2	11.1	6.4	7.1	7.7	8.4	9.2	9.9	10.9
30	7.0	7.7	8.4	9.1	9.8	10.6	11.5	6.7	7.4	8.1	8.8	9.6	10.3	11.3
31	7.4	8.1	8.8	9.5	10.2	11.0	11.9	7.1	7.8	8.5	9.2	10.0	10.8	11.7
32	7.8	8.5	9.2	9.9	10.7	11.4	12.3	7.6	8.3	8.9	9.7	10.4	11.2	12.1
33	8.2	9.0	9.6	10.4	11.1	11.9	12.8	8.0	8.7	9.4	10.1	10.9	11.7	12.6
34	8.7	9.4	10.1	10.8	11.6	12.4	13.2	8.5	9.2	9.9	10.6	11.4	12.2	13.1
35	9.2	9.9	10.6	11.3	12.1	12.8	13.7	9.0	9.7	10.4	11.1	11.9	12.7	13.6
36	9.7	10.4	11.1	11.8	12.6	13.3	14.2	9.5	10.2	10.9	11.6	12.4	13.1	14.0
37	10.2	10.9	11.6	12.3	13.1	13.8	14.6	10.0	10.7	11.4	12.1	12.9	13.6	14.4
38	10.7	11.4	12.1	12.8	13.5	14.2	15.1	10.5	11.2	11.8	12.6	13.3	14.1	14.9
39	11.1	11.8	12.4	13.1	13.9	14.6	15.4	10.9	11.6	12.2	12.9	13.7	14.4	15.2
40	11.4	12.1	12.7	13.4	14.1	14.8	15.6	11.3	11.9	12.5	13.2	14.0	14.7	15.4
41	11.7	12.3	12.9	13.6	14.3	15.0	15.8	11.5	12.1	12.8	13.5	14.2	14.9	15.6
42	11.9	12.5	13.1	13.8	14.5	15.2	15.9	11.8	12.4	13.0	13.7	14.4	15.0	15.8

**Table 8:** Percentile reference values of birth PI (in kg/m^3^) for Chinese newborn infants aged 24–42 weeks.

GA (weeks)	Male							Female						
	P_3_	P_10_	P_25_	P_50_	P_75_	P_90_	P_97_	P_3_	P_10_	P_25_	P_50_	P_75_	P_90_	P_97_
24	16.3	18.0	19.8	21.9	24.2	26.5	29.2	15.6	17.3	19.1	21.2	23.5	25.8	28.3
25	16.4	18.1	19.9	22.0	24.2	26.6	29.3	15.8	17.5	19.3	21.4	23.7	25.9	28.4
26	16.5	18.2	20.0	22.1	24.3	26.6	29.3	16.1	17.8	19.5	21.6	23.8	26.0	28.5
27	16.7	18.4	20.2	22.2	24.4	26.7	29.3	16.3	18.0	19.7	21.8	24.0	26.2	28.6
28	16.9	18.6	20.3	22.4	24.5	26.8	29.3	16.6	18.2	20.0	22.0	24.2	26.4	28.8
29	17.2	18.9	20.6	22.5	24.7	26.9	29.4	16.9	18.5	20.2	22.2	24.4	26.5	28.9
30	17.5	19.1	20.8	22.7	24.8	27.0	29.4	17.2	18.8	20.5	22.5	24.6	26.7	29.1
31	17.8	19.4	21.1	23.0	25.0	27.1	29.5	17.6	19.2	20.8	22.8	24.9	26.9	29.3
32	18.2	19.8	21.4	23.3	25.2	27.2	29.5	18.0	19.6	21.2	23.1	25.1	27.2	29.5
33	18.7	20.2	21.8	23.6	25.5	27.4	29.6	18.5	20.0	21.6	23.5	25.4	27.4	29.6
34	19.2	20.7	22.2	23.9	25.8	27.6	29.7	19.0	20.5	22.1	23.8	25.7	27.6	29.8
35	19.8	21.2	22.7	24.3	26.1	27.8	29.8	19.6	21.1	22.5	24.2	26.0	27.9	29.9
36	20.4	21.8	23.2	24.7	26.4	28.0	29.9	20.3	21.7	23.1	24.7	26.4	28.1	30.0
37	21.1	22.4	23.7	25.2	26.7	28.3	30.0	20.9	22.3	23.6	25.1	26.7	28.3	30.2
38	21.7	23.0	24.2	25.6	27.1	28.6	30.3	21.6	22.9	24.1	25.6	27.1	28.6	30.3
39	22.2	23.5	24.7	26.0	27.5	28.9	30.5	22.1	23.4	24.6	26.0	27.5	28.9	30.6
40	22.6	23.8	25.1	26.4	27.8	29.2	30.8	22.5	23.7	24.9	26.3	27.7	29.2	30.8
41	22.9	24.1	25.4	26.7	28.1	29.4	31.1	22.8	24.0	25.2	26.6	28.0	29.5	31.1
42	23.2	24.5	25.7	27.0	28.3	29.7	31.3	23.1	24.3	25.5	26.8	28.3	29.7	31.4

### Comparison of fitted centile curves and observed empirical values

**Figure 1:**
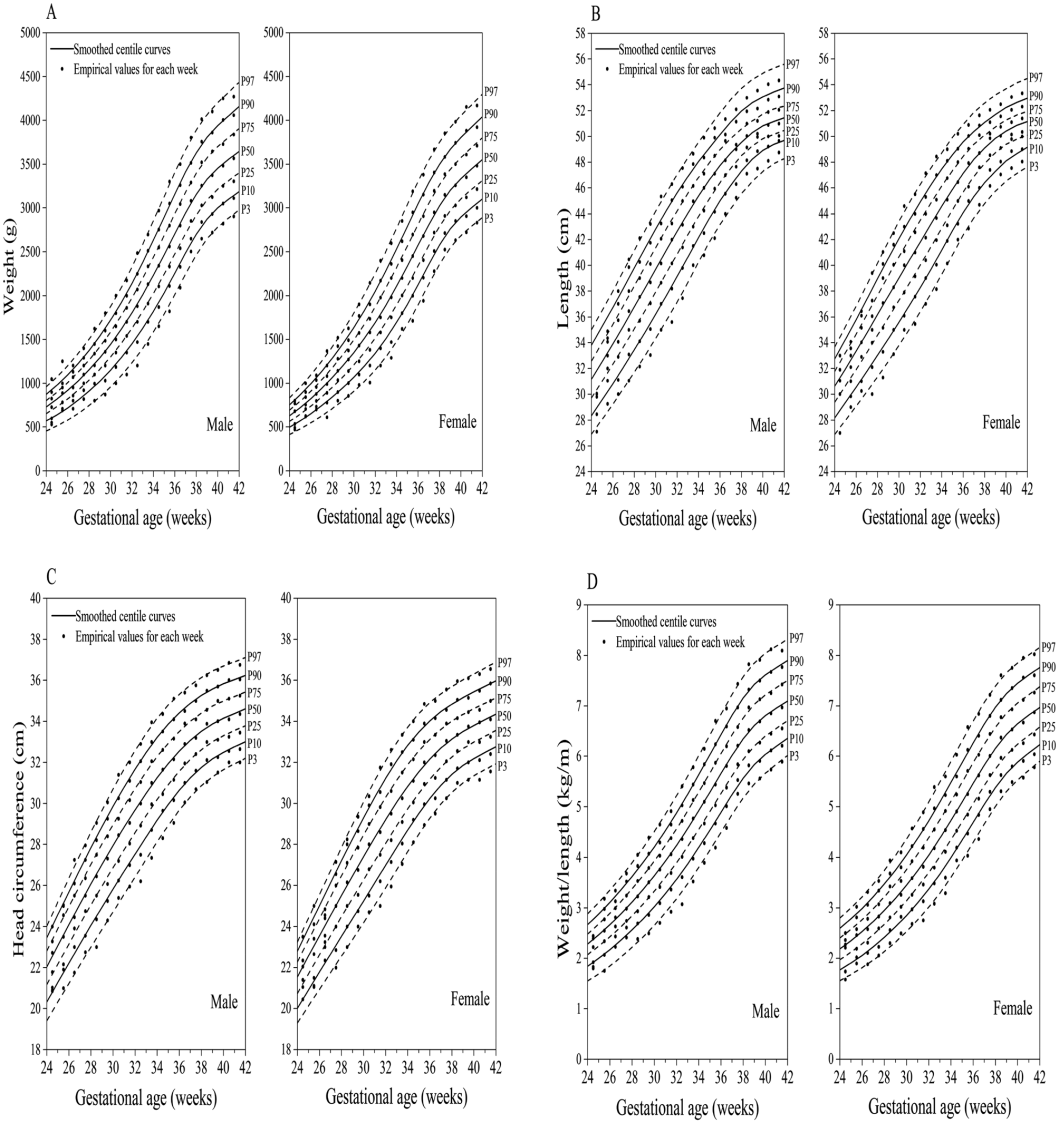

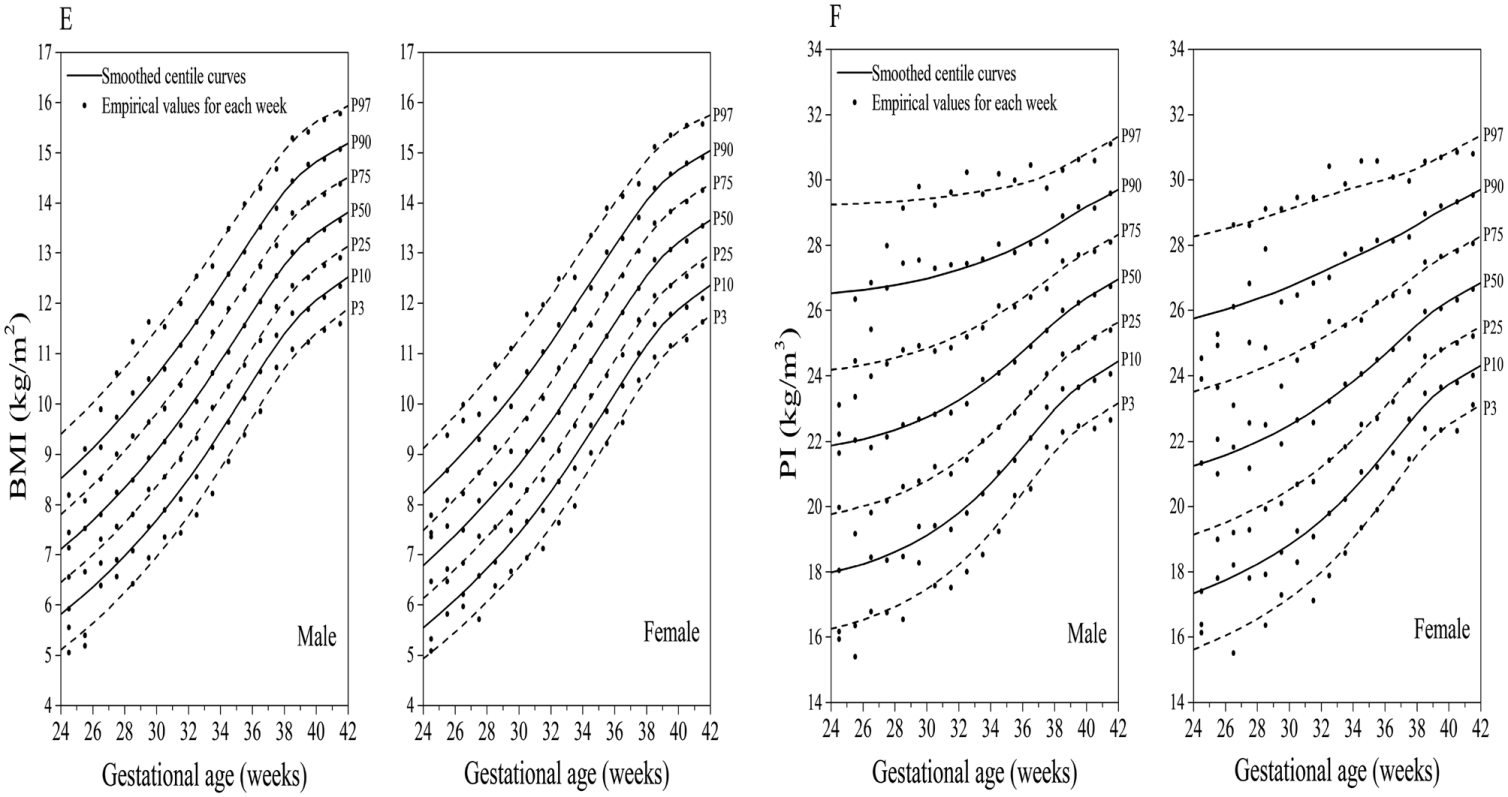
Comparison of the P_3_, P_10_, P_25_, P_50_, P_75_, P_90_, and P_97_ of smoothed fitted curves and observed empirical values of birth weight (**A**), length (**B**), head circumference (**C**), weight/length (**D**), BMI (**E**), and PI (**F**) in China.

Figure 1A to F demonstrates the comparison of smoothed fitted centiles and observed empirical values of birth weight, length, head circumference, weight/length, BMI, and PI by sex and GA, showing almost identical values with very few exceptions at the lower end of GA distribution where only a small number of individual measures could be obtained, i.e., at 24–26 weeks of gestation.

### Comparison of the China standards with the INTERGROWTH-21st standards

**Figure 2:**
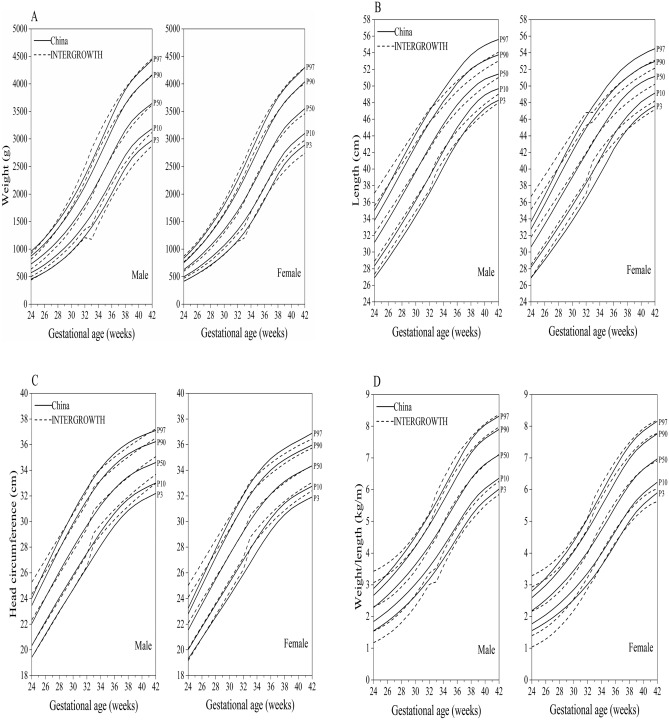
Comparison of the P_3_, P_10_, P_50_, P_90_, and P_97_ curves of birth weight (**A**), length (**B**), head circumference (**C**), and weight/length (**D**) in China with the INTERGROWTH-21st standards.

Overall, the percentile curves of birth weight, length, head circumference, and weight/length in China presented similar growth trajectories with the INTERGROWTH-21st standards, but also expressed distinct differences for length at 37–42 weeks and weight/length at 24–32 weeks (Fig. 2A to D).

### Comparison of the China standards with the new US curves

**Figure 3:**
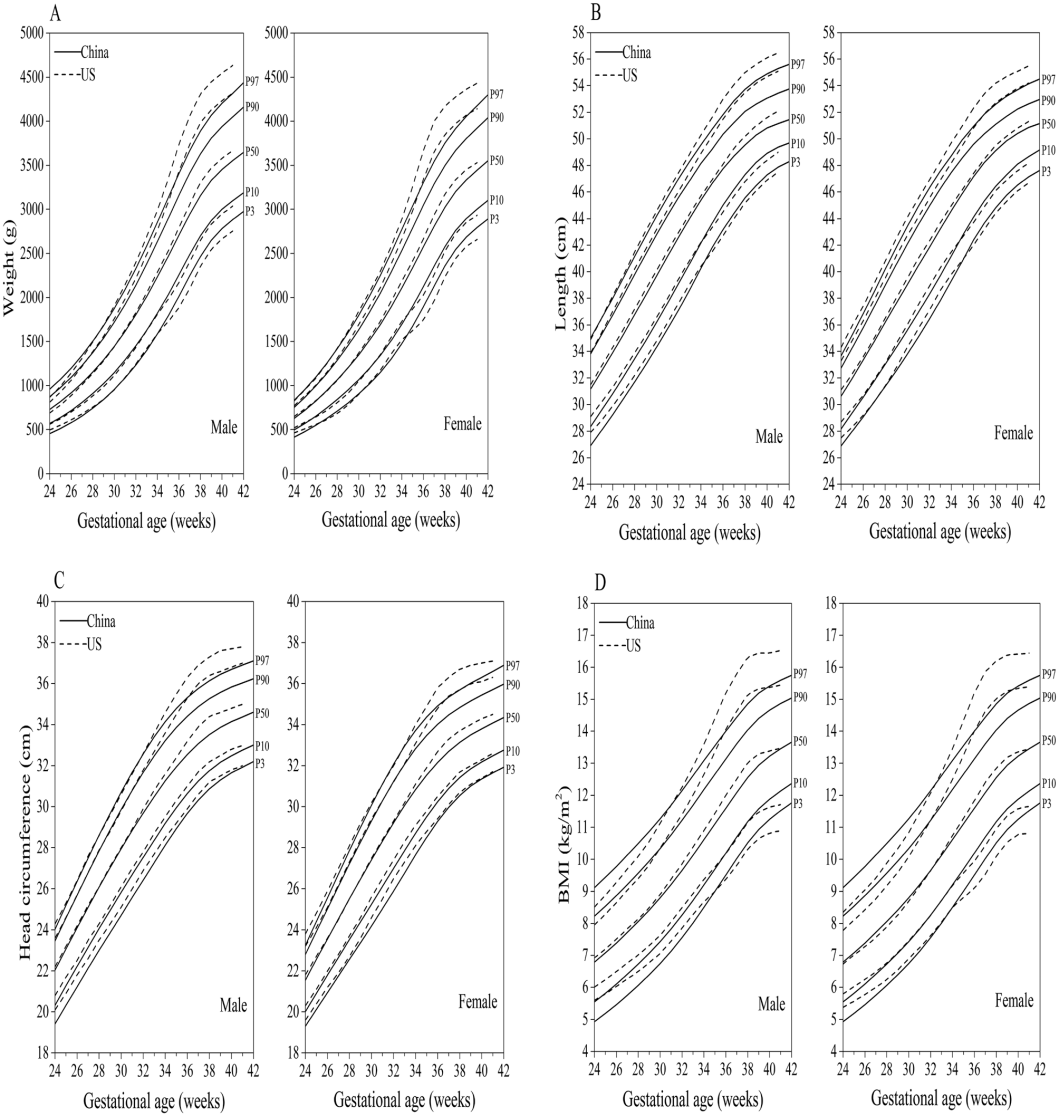
Comparison of the P_3_, P_10_, P_50_, P_90_, and P_97_ curves of birth weight (**A**), length (**B**), head circumference (**C**), and BMI (**D**) in China with the new US curves.

There are small differences for the percentile curves of birth weight, length, and head circumference at 24–36 weeks between China and US curves, but considerable differences at 37–41 weeks, especially at the upper centiles (e.g., 90th); and there is a large disparity for birth BMI from 24 to 41 weeks (Fig. 3A to D).

## Discussion

The reference sample of our standards derive from a population-based survey of newborns from economically developed urban areas with strict criteria for inclusion, such as single live birth, naturally conception, and health condition of both the mother and the newborn. The shapes of growth curves based on low-risk pregnancies with a normal outcome may differ from those of growth curves generated from more conventional data sets that include both low- and high-risk pregnancies. These differences mainly reflect the variations in the distance between the lowermost and uppermost centiles (e.g., 3rd and 97th). For example, the distance in our weight curves from low-risk individuals was shorter compared to other weight curves from Chinese routine monitoring data that included both low- and high-risk individuals^11,12^. Similar to our observations, new and improved Dutch birth weight percentile curves based on data from low-risk pregnancies displayed lower range/variation, which proved to be more effective in identifying clinically important risk SGA infants^2,22^.

The health of the mother and the newborn is the foundation of sustainable development for individuals, families, and societies as it is closely linked with health throughout life^23^. Assessment of growth and nutrition of the neonates is essential for a positive outcome in later life. However, no single anthropometric measure fully reflects growth, development and health of newborn babies, so we established percentile curves of multiple anthropometric measures, with each measure revealing distinct relationships with specific health risks or diseases. Birth weight is typically used to define the classification of newborn size as small, appropriate, or large for a specified GA at birth^24^. Birth length is helpful in evaluating whether postnatal catch-up growth is appropriate^25,26^. Birth head circumference reflects intrauterine brain development and predicts the prognosis of nervous system development^27^. A consensus was reached on the definition of growth restriction as birth weight < 3rd percentile or at least 3 out of 5 of the following: birth weight < 10th percentile, length < 10th percentile, head circumference < 10th percentile, prenatal diagnosis of fetal growth restriction, and maternal pregnancy complications^28^. Traditional classification based on birth weight centiles for GA does not reflect body fat in both term and preterm newborns^29^, while weight/length greatly aids in predicting newborn fat mass and fat-free mass as well as body proportionality^15,30^. BMI is useful for measuring body proportionality for newborn infants^30–32^. PI is a customary measure to evaluate whether abnormalities in growth in preterm infants are symmetric or asymmetric^33–35^. In sum, our established reference values of six indicators can provide more tools for growth and nutrition assessment (e.g., frequently using weight, length and head circumference) and nutrition assessment/body proportionality (e.g., frequently using weight/length, BMI and PI) in neonatal clinical practice.

Our study has several strengths. First, our standards were based on low-risk pregnancies with a normal outcome. Second, besides the commonly used weight, length, and head circumference, we also established percentile curves of weight/length, BMI, and PI that help assess whether abnormal babies in growth are symmetric or asymmetric. Under strict exclusion criteria, we included a relatively large sample size of preterm infants that guaranteed more reliable percentile curves; however, the screening efficacy of this set of new standards for SGA or LGA and body proportionality still needs to be further validated and evaluated.

Based on a contemporary, large-scaled, population-based cross-sectional nationally representative sample from low-risk pregnancies with a normal outcome that represents optimal intrauterine growth, we developed a set of neonatal growth standards for 24–42 weeks of gestation, including reference values of six anthropometric indicators that can provide more tools for growth and nutrition assessment and body proportionality in neonatal clinical practice. In addition, our study aids in better understanding the differences in the shapes of growth curves between based on data from low-risk pregnancies only or from mixed low- and high-risk pregnancies.

## Data Availability

The datasets generated during and/or analysed during the current study are not publicly available due to the confidential policy of our institute and hospital but are available from the corresponding author on reasonable request.

## References

[1] Villar J (2014). International standards for newborn weight, length, and head circumference by gestational age and sex: The Newborn Cross-Sectional Study of the INTERGROWTH-21st Project. Lancet.

[2] Hoftiezer L (2019). From population reference to national standard: New and improved birthweight charts. Am. J. Obstet. Gynecol..

[3] Cole TJ, Williams AF, Wright CM, RCPCH Growth Chart Expert Group (2011). Revised birth centiles for weight, length and head circumference in the UK-WHO growth charts. Ann. Hum. Biol..

[4] Olsen IE, Groveman SA, Lawson ML, Clark RH, Zemel BS (2010). New intrauterine growth curves based on United States data. Pediatrics.

[5] Bertino E (2010). Neonatal anthropometric charts: The Italian neonatal study compared with other European studies. J. Pediatr. Gastroenterol. Nutr..

[6] Fenton TR, Kim JH (2013). A systematic review and meta-analysis to revise the Fenton growth chart for preterm infants. BMC Pediatr..

[7] Kiserud T (2018). The World Health Organization fetal growth charts: Concept, findings, interpretation, and application. Am. J. Obstet. Gynecol..

[8] Zhang J, Merialdi M, Platt LD, Kramer MS (2010). Defining normal and abnormal fetal growth: Promises and challenges. Am. J. Obstet. Gynecol..

[9] Cole TJ (2012). The development of growth references and growth charts. Ann. Hum. Biol..

[10] Zhang B, Feng Z, Zhang L (1988). An investigation on the physical development of neonates of various gestational age in 15 cities of China. Chin. J. Pediatr..

[11] Zhu L (2015). Chinese neonatal birth weight curve for different gestational age. Chin. J. Pediatr..

[12] Dai L (2014). Birth weight reference percentiles for Chinese. PLoS ONE.

[13] Yao F, Miao H, Li B, Wu Y, Zhao Q (2018). New birthweight percentiles by sex and gestational age in Southern China and its comparison with the INTERGROWTH-21st Standard. Sci. Rep..

[14] Villar J (2016). INTERGROWTH-21st very preterm size at birth reference charts. Lancet.

[15] Villar J (2017). Body composition at birth and its relationship with neonatal anthropometric ratios: The newborn body composition study of the INTERGROWTH-21st project. Pediatr. Res..

[16] Olsen IE (2015). BMI curves for preterm infants. Pediatrics.

[17] Zhang YQ (2017). The 5th national survey on the physical growth and development of children in the nine cities of China: Anthropometric measurements of Chinese children under 7 years in 2015. Am. J. Phys. Anthropol..

[18] Zong XN, Li H (2020). Establishment of growth standards for Chinese newborns by gestational ages: Study design and statistical methods. Chin. J. Evid. Based Pediatr..

[19] Rigby RA, Stasinopoulos DM (2014). Automatic smoothing parameter selection in GAMLSS with an application to centile estimation. Stat. Methods Med. Res..

[20] Royston P, Wright EM (2000). Goodness-of-fit statistics for age-specific reference intervals. Stat. Med..

[21] van Buuren S, Fredriks M (2001). Worm plot: A simple diagnostic device for modelling growth reference curves. Stat. Med..

[22] Hoftiezer L (2016). Defining small-for-gestational-age: Prescriptive versus descriptive birthweight standards. Eur. J. Pediatr..

[23] Qiao J (2021). A Lancet Commission on 70 years of women's reproductive, maternal, newborn, child, and adolescent health in China. Lancet.

[24] Schlaudecker EP (2017). Small for gestational age: Case definition & guidelines for data collection, analysis, and presentation of maternal immunisation safety data. Vaccine.

[25] Taal HR (2013). Small and large size for gestational age at birth, infant growth, and childhood overweight. Obesity (Silver Spring).

[26] Karlberg J, Albertsson-Wikland K (1995). Growth in full-term small-for-gestational-age infants: From birth to final height. Pediatr. Res..

[27] Barbier A (2013). New reference curves for head circumference at birth, by gestational age. Pediatrics.

[28] Beune IM (2018). Consensus Based Definition of Growth Restriction in the Newborn. J. Pediatr..

[29] Schmelzle HR, Quang DN, Fusch G, Fusch C (2007). Birth weight categorization according to gestational age does not reflect percentage body fat in term and preterm newborns. Eur. J. Pediatr..

[30] Davidson S (2011). Body mass index and weight-for-length ratio references for infants born at 33–42 weeks gestation: A new tool for anthropometric assessment. Clin. Nutr..

[31] Ferguson AN (2018). BMI is a better body proportionality measure than the ponderal index and weight-for-length for preterm infants. Neonatology.

[32] Williamson AL (2018). Longitudinal BMI growth curves for surviving preterm NICU infants based on a large US sample. Pediatrics.

[33] Villar J (1990). The differential neonatal morbidity of the intrauterine growth retardation syndrome. Am. J. Obstet. Gynecol..

[34] Cole TJ, Henson GL, Tremble JM, Colley NV (1997). Birthweight for length: Ponderal index, body mass index or Benn index?. Ann. Hum. Biol..

[35] Landmann E, Reiss I, Misselwitz B, Gortner L (2006). Ponderal index for discrimination between symmetric and asymmetric growth restriction: Percentiles for neonates from 30 weeks to 43 weeks of gestation. J. Matern. Fetal Neonatal Med..

